# Genetic Prediction of Kidney Function: Why Measured GFR Matters

**DOI:** 10.1016/j.ekir.2025.103724

**Published:** 2025-12-13

**Authors:** Atlas Khan

**Affiliations:** 1 Division of Nephrology, Department of Medicine, Vagelos College of Physicians and Surgeons, Columbia University, New York, New York, USA

See Clinical Research on Page 196

Chronic kidney disease is a major global health challenge, affecting an estimated 10% to 15% of the population and contributing significantly to morbidity and mortality worldwide.^1^ Chronic kidney disease exhibits substantial heritability, with a complex genetic architecture shaped by both rare mutations and polygenic contributions from numerous common variants.^2^, ^3^, ^4^ Genome-wide association studies (GWAS) have identified hundreds of loci associated with kidney function, enabling the development of genome-wide polygenic scores (GPS) that aggregate the effects of thousands to millions of single-nucleotide polymorphisms to quantify inherited risk.^5^^,^^6^

Despite these advances, nearly all kidney GPS, including those developed by our group and others, rely on GWAS of estimated glomerular filtration rate (GFR, eGFR).^7^ eGFR, derived from serum creatinine or cystatin C, is chosen for its feasibility in large cohorts but remains an imperfect proxy for true kidney function. Creatinine and cystatin C are influenced by multiple non-GFR factors, including muscle mass, inflammation, tubular secretion, medications, and metabolic status.^8^ Consequently, GWAS based on eGFR capture a mixture of genuine GFR-related effects and confounding non-GFR signals, potentially distorting the genetic architecture reflected in GPS.

In the article by, Eriksen *et al*.^9^ directly address this limitation by evaluating eGFR-derived GPS against rigorously measured GFR (mGFR) using iohexol clearance in the Renal Iohexol Clearance Survey cohort.^10^ Among 1492 participants with repeated mugger measurements over 11 years, the authors compared the genetic architecture of mgrs. with several eGFR-based phenotypes (eGFRcr, eGFRcys, and eGFRcr-cys). Their analyses revealed consistently larger genetic effect sizes and heritability estimates for mGFR compared with any eGFR metric, highlighting that current eGFR-based GPS are influenced by non-GFR determinants of creatinine and cystatin C.

Differences in the biology of these biomarkers, creatinine, iohexol, and cystatin C, likely underlie these discrepancies. Specifically, mGFR and eGFRcys showed nearly identical results across the tested GPS but consistently lower predictive performance than eGFRcr- or combined eGFRcr-cys-based scores, suggesting that eGFR-derived GPS include variants associated with non-GFR determinants. These findings underscore the limitations of relying solely on eGFR-derived GWAS and GPS to understand the genetics of kidney function and support the need for validation using more precise measures such as mGFR.

The strength of this study lies in its rigorous design. Renal Iohexol Clearance Survey is the only population-based cohort with repeated, gold-standard iohexol GFR measurements paired with dense genome-wide genotyping, allowing a rare, direct comparison of the genetics of measured versus estimated kidney function. High-quality individual-level genotype data further strengthen the analyses. Nevertheless, the study has limitations. Its modest sample size constrains statistical power for variant-level characterization of eGFR bias and reduces precision in heritability estimates. The cohort was relatively homogeneous, middle-aged adults of European ancestry without diabetes, cardiovascular disease, or kidney disease, limiting generalizability to populations with more comorbidities, where discrepancies between eGFR and mGFR may be larger. Finally, though the results suggest that mGFR captures a stronger and less confounded genetic signal, the absence of comparable cohorts prevented the construction or external validation of an mGFR-based GPS.

These findings have important implications for clinical practice and the use of genetic risk scores. GPS derived from eGFR-based GWAS may underestimate true genetic risk, particularly in individuals with atypical biomarker profiles such as sarcopenia, obesity, chronic inflammation, or polypharmacy. The stronger genetic signal captured by mGFR suggests that, as more feasible measures of true GFR become available, they could improve genetic stratification, enable earlier identification of high-risk individuals, and enhance the precision of chronic kidney disease prediction. Although routine clinical measurement of mGFR remains impractical because of cost and logistical challenges, this study underscores the need for improved biomarkers and genetic risk tools that more faithfully reflect true kidney function. Incorporating precise phenotyping into genetic studies has the potential to further increase the clinical utility of polygenic risk scores in personalized patient care.

### Funding

This work was supported by the 10.13039/100000062National Institute of Diabetes and Digestive and Kidney Diseases (NIDDK) under grants K25DK128563-05 and R03DK144285-01. The findings and conclusions in this report are those of the authors and do not necessarily represent the official position of the funding institution.
